# (*S*)-1-[3,5-Bis­(trifluoro­meth­yl)phen­yl]-*N*-methylethyl­amine–(*R*)-2-hydroxy­butane­dioic acid (1/1)

**DOI:** 10.1107/S1600536808043201

**Published:** 2008-12-24

**Authors:** Hai-Bin Zhu, Jun-Feng Ji, Hai Wang

**Affiliations:** a School of Chemistry and Chemical Engineering, Southeast University, Nanjing, People’s Republic of China

## Abstract

In the title compound, C_11_H_11_F_6_N·C_4_H_6_O_5_, a key inter­mediate in the synthesis of the NK1 receptor antagonist of casopitant, the F atoms of the trifluoro­methyl groups are disordered over two sites with equal occupancies. In the crystal, the components are linked by bifurcated N—H⋯(O,O) hydrogen bonds.

## Related literature

The title compound is a key intermediate for the synthesis of casopitant, which is an NK1 receptor antagonist (Humphrey, 2003 ▶) for the treatment of chemotheraphy-induced nausea and vomiting (CINV) (Lohr, 2008 ▶).
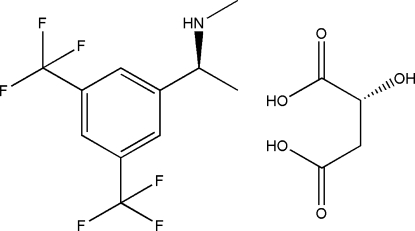


## Experimental

### 

#### Crystal data

C_11_H_11_F_6_N·C_4_H_6_O_5_
*M* *_r_* = 405.30
Monoclinic, 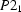

*a* = 6.6770 (13) Å
*b* = 8.4510 (17) Å
*c* = 16.366 (3) Å
β = 100.05 (3)°
*V* = 909.3 (3) Å^3^
*Z* = 2
Mo *K*α radiation
μ = 0.15 mm^−1^
*T* = 298 (2) K
0.30 × 0.10 × 0.10 mm

#### Data collection

Enraf–Nonius CAD-4 diffractometer
Absorption correction: ψ scan (North *et al.*, 1968 ▶) *T*_min_ = 0.957, *T*_max_ = 0.985
1915 measured reflections
1757 independent reflections
1067 reflections with *I* > 2σ(*I*)
*R*_int_ = 0.058
3 standard reflections every 200 reflections intensity decay: 1%

#### Refinement

*R*[*F* ^2^ > 2σ(*F* ^2^)] = 0.063
*wR*(*F* ^2^) = 0.154
*S* = 1.00
1757 reflections
220 parameters
2 restraints
H-atom parameters constrained
Δρ_max_ = 0.18 e Å^−3^
Δρ_min_ = −0.27 e Å^−3^

### 

Data collection: *CAD-4 Software* (Enraf–Nonius,1989 ▶); cell refinement: *CAD-4 Software*; data reduction: *XCAD4* (Harms & Wocadlo, 1995 ▶); program(s) used to solve structure: *SHELXS97* (Sheldrick, 2008 ▶); program(s) used to refine structure: *SHELXL97* (Sheldrick, 2008 ▶); molecular graphics: *SHELXTL* (Sheldrick, 2008 ▶); software used to prepare material for publication: *SHELXL97*.

## Supplementary Material

Crystal structure: contains datablocks I, global. DOI: 10.1107/S1600536808043201/at2692sup1.cif

Structure factors: contains datablocks I. DOI: 10.1107/S1600536808043201/at2692Isup2.hkl

Additional supplementary materials:  crystallographic information; 3D view; checkCIF report

## Figures and Tables

**Table 1:** Hydrogen-bond geometry (Å, °)

*D*—H⋯*A*	*D*—H	H⋯*A*	*D*⋯*A*	*D*—H⋯*A*
N—H0*A*⋯O4^i^	0.86	2.37	2.914 (7)	122
N—H0*A*⋯O3^ii^	0.86	2.20	2.887 (7)	137

Symmetry codes: (i) 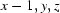; (ii) 
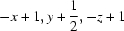
.

**Fig. 1.**
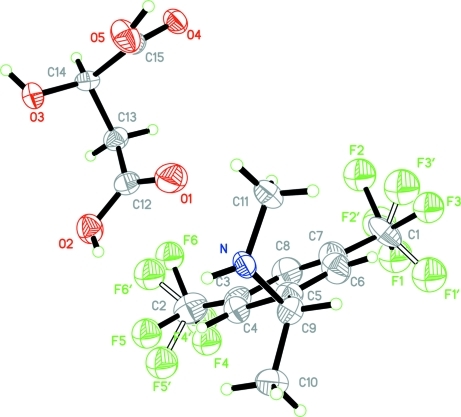
The molecular structure of the title compound, drawn with 30% probability ellipsoids.

## supplementary crystallographic information

### Comment

The title compound, C_11_H_11_F_6_N.C_4_H_6_O_5_, is a key intermediate for the synthesis of casopitant, which is an NK1 receptor antagonist (Humphrey, 2003) for the treatment of chemotheraphy-induced nausea and vomiting (CINV) (Lohr, 2008).

The molecular structure of the title compound is shown in Fig.1. The F atoms of the trifluoromethyl group are disordered over two sites in a 0.50:0.50 ratio. N—H···O hydrogen bonding interactions occur between (*S*)-1-(3, 5-bis(trifluoromethyl)-phenyl)ethylamine *N*-monomethyl and (*R*)-2-hydroxybutanedioic acid (Table 1).

### Experimental

To a solution of 3, 5-bis(trifluoromethyl)-phenyl)ethylamine *N*-monomethyl (2.71 g, 10 mmol) in EtOAc (25 ml), (*R*)-2-hydroxybutanedioic acid (1.34,10 mmol) was added portionwise. The suspension was stirred for 2h at 298 K, then for 3 h at 273 K. The suspension was filtered and the cake was washed with EtOAc (20 ml). The solid was dried under vacucum obtaining the crude title compound (1.48 g). Single crystal of the title compound suitable for X-ray diffraction was obtained by slow evaporation of the EtOAc solution of the title compound.

### Refinement

All H atoms were positoned geometrically and allowed to ride on their parent atoms, with C—H = 0.93Å for aromatic H atoms, 0.96Å for methyl H atoms, 0.97Å for methylene H atoms, 0.98Å for methine H atoms and O—H = 0.82 Å, N—H = 0.86 Å, respectively. [*U*_iso_ (H) = 1.2*U*_eq_(C) for aromatic, methylene and methine; *U*_iso_(H) = 1.5 *U*_eq_(C) for methyl, *U*_iso_(H) = 1.2*U*_eq_(N); *U*_iso_(H) = 1.5 *U*_eq_(O).]

### Crystal data

**Table:**

C_11_H_11_F_6_N·C_4_H_6_O_5_	*F*(000) = 416
*M**_r_* = 405.30	*D*_x_ = 1.480 Mg m^−^^3^
Monoclinic, *P*2_1_	Mo *K*α radiation, λ = 0.71073 Å
Hall symbol: P 2yb	Cell parameters from 25 reflections
*a* = 6.6770 (13) Å	θ = 9–12°
*b* = 8.4510 (17) Å	µ = 0.15 mm^−^^1^
*c* = 16.366 (3) Å	*T* = 298 K
β = 100.05 (3)°	Needle, colourless
*V* = 909.3 (3) Å^3^	0.30 × 0.10 × 0.10 mm
*Z* = 2	

### Data collection

**Table:**

Enraf–Nonius CAD-4 diffractometer	1067 reflections with *I* > 2σ(*I*)
Radiation source: fine-focus sealed tube	*R*_int_ = 0.058
graphite	θ_max_ = 25.3°, θ_min_ = 1.3°
ω/2θ scans	*h* = 0→7
Absorption correction: ψ scan (North *et al.*, 1968)	*k* = 0→10
*T*_min_ = 0.957, *T*_max_ = 0.985	*l* = −19→19
1915 measured reflections	3 standard reflections every 200 reflections
1757 independent reflections	intensity decay: 1%

### Refinement

**Table:**

Refinement on *F*^2^	Primary atom site location: structure-invariant direct methods
Least-squares matrix: full	Secondary atom site location: difference Fourier map
*R*[*F*^2^ > 2σ(*F*^2^)] = 0.063	Hydrogen site location: inferred from neighbouring sites
*wR*(*F*^2^) = 0.154	H-atom parameters constrained
*S* = 1.00	*w* = 1/[σ^2^(*F*_o_^2^) + (0.060*P*)^2^ + 0.3*P*] where *P* = (*F*_o_^2^ + 2*F*_c_^2^)/3
1757 reflections	(Δ/σ)_max_ < 0.001
220 parameters	Δρ_max_ = 0.18 e Å^−^^3^
2 restraints	Δρ_min_ = −0.27 e Å^−^^3^

### Special details

**Table:**

Geometry. All e.s.d.'s (except the e.s.d. in the dihedral angle between two l.s. planes) are estimated using the full covariance matrix. The cell e.s.d.'s are taken into account individually in the estimation of e.s.d.'s in distances, angles and torsion angles; correlations between e.s.d.'s in cell parameters are only used when they are defined by crystal symmetry. An approximate (isotropic) treatment of cell e.s.d.'s is used for estimating e.s.d.'s involving l.s. planes.
Refinement. Refinement of *F*^2^ against ALL reflections. The weighted *R*-factor *wR* and goodness of fit *S* are based on *F*^2^, conventional *R*-factors *R* are based on *F*, with *F* set to zero for negative *F*^2^. The threshold expression of *F*^2^ > σ(*F*^2^) is used only for calculating *R*-factors(gt) *etc*. and is not relevant to the choice of reflections for refinement. *R*-factors based on *F*^2^ are statistically about twice as large as those based on *F*, and *R*- factors based on ALL data will be even larger.

### Fractional atomic coordinates and isotropic or equivalent isotropic displacement parameters (Å^2^)

**Table:**

	*x*	*y*	*z*	*U*_iso_*/*U*_eq_	Occ. (<1)
F1	0.9058 (17)	0.4852 (16)	0.9973 (8)	0.112	0.50
F3	0.9270 (13)	0.6270 (13)	0.8772 (6)	0.083	0.50
F2	1.0892 (15)	0.4013 (14)	0.9084 (6)	0.101	0.50
F1'	0.7985 (16)	0.6070 (16)	0.9651 (7)	0.111	0.50
F2'	1.000 (2)	0.4376 (16)	0.9758 (7)	0.111	0.50
F3'	0.9991 (17)	0.5461 (16)	0.8769 (7)	0.115	0.50
F4	0.5055 (13)	−0.0158 (14)	0.9651 (7)	0.085	0.50
F5	0.3781 (15)	−0.0926 (12)	0.8429 (6)	0.080	0.50
F6	0.6975 (13)	−0.0918 (12)	0.8664 (6)	0.078	0.50
F4'	0.5831 (15)	−0.0443 (15)	0.9665 (8)	0.097	0.50
F5'	0.3083 (15)	−0.0556 (14)	0.8707 (6)	0.095	0.50
F6'	0.6174 (16)	−0.1374 (14)	0.8609 (7)	0.093	0.50
N	0.3387 (7)	0.3503 (6)	0.6045 (3)	0.0464 (12)	
H0A	0.2534	0.2888	0.5744	0.056*	
C1	0.8859 (12)	0.4933 (11)	0.9164 (5)	0.081	
C2	0.5244 (14)	−0.0104 (14)	0.8816 (5)	0.094 (3)	
C3	0.5326 (11)	0.1454 (10)	0.8548 (4)	0.063 (2)	
C4	0.4157 (10)	0.2037 (8)	0.7869 (4)	0.0543 (17)	
H4A	0.3136	0.1408	0.7574	0.065*	
C5	0.4445 (9)	0.3611 (9)	0.7587 (4)	0.0527 (16)	
C6	0.5931 (11)	0.4465 (10)	0.8000 (4)	0.072 (2)	
H6A	0.6137	0.5488	0.7821	0.087*	
C7	0.7249 (13)	0.3856 (12)	0.8723 (5)	0.080 (2)	
C8	0.6962 (12)	0.2426 (12)	0.8978 (5)	0.079 (3)	
H8A	0.7817	0.2026	0.9441	0.095*	
C9	0.3017 (8)	0.4239 (8)	0.6855 (3)	0.0482 (15)	
H9A	0.3274	0.5377	0.6823	0.058*	
C10	0.0783 (9)	0.4033 (9)	0.6903 (4)	0.0646 (19)	
H10A	0.0507	0.4508	0.7405	0.097*	
H10B	0.0461	0.2926	0.6902	0.097*	
H10C	−0.0033	0.4535	0.6433	0.097*	
C11	0.5373 (9)	0.3961 (9)	0.5832 (4)	0.0606 (18)	
H11A	0.5542	0.3455	0.5323	0.091*	
H11B	0.6447	0.3636	0.6269	0.091*	
H11C	0.5419	0.5088	0.5766	0.091*	
O1	0.6777 (8)	0.0742 (7)	0.6474 (4)	0.0869 (18)	
O2	0.5220 (7)	−0.1471 (5)	0.6689 (3)	0.0652 (13)	
H2A	0.4226	−0.0912	0.6526	0.098*	
O3	0.8263 (6)	−0.2740 (5)	0.5576 (3)	0.0579 (12)	
H3A	0.8825	−0.3437	0.5351	0.087*	
O4	1.1965 (6)	0.0264 (5)	0.6157 (3)	0.0540 (11)	
O5	0.9516 (7)	0.0076 (6)	0.5086 (3)	0.0678 (15)	
H5A	1.0184	0.0743	0.4888	0.102*	
C12	0.6858 (11)	−0.0671 (9)	0.6676 (4)	0.0551 (17)	
C13	0.8797 (9)	−0.1508 (8)	0.6891 (4)	0.0522 (15)	
H13A	0.8587	−0.2481	0.7179	0.063*	
H13B	0.9744	−0.0857	0.7265	0.063*	
C14	0.9715 (8)	−0.1898 (7)	0.6122 (3)	0.0428 (14)	
H14A	1.0894	−0.2588	0.6292	0.051*	
C15	1.0461 (9)	−0.0360 (7)	0.5766 (4)	0.0464 (15)	

### Atomic displacement parameters (Å^2^)

**Table:**

	*U*^11^	*U*^22^	*U*^33^	*U*^12^	*U*^13^	*U*^23^
F1	0.112	0.112	0.112	0.000	0.020	0.000
F3	0.083	0.083	0.083	0.000	0.015	0.000
F2	0.101	0.101	0.101	0.000	0.018	0.000
F1'	0.111	0.111	0.111	0.000	0.019	0.000
F2'	0.111	0.111	0.111	0.000	0.019	0.000
F3'	0.115	0.115	0.115	0.000	0.020	0.000
F4	0.085	0.085	0.085	0.000	0.015	0.000
F5	0.080	0.080	0.080	0.000	0.014	0.000
F6	0.078	0.078	0.078	0.000	0.014	0.000
F4'	0.097	0.097	0.097	0.000	0.017	0.000
F5'	0.095	0.095	0.095	0.000	0.017	0.000
F6'	0.093	0.093	0.093	0.000	0.016	0.000
N	0.050 (3)	0.044 (3)	0.047 (3)	−0.015 (3)	0.010 (2)	−0.004 (3)
C1	0.072	0.100	0.067	−0.033	−0.003	0.033
C2	0.097 (7)	0.114 (8)	0.069 (6)	0.014 (7)	0.012 (5)	0.000 (6)
C3	0.063 (4)	0.065 (5)	0.059 (4)	0.016 (4)	0.005 (4)	0.000 (4)
C4	0.055 (4)	0.058 (4)	0.048 (3)	−0.002 (4)	0.002 (3)	0.002 (3)
C5	0.053 (4)	0.065 (5)	0.042 (3)	−0.004 (4)	0.013 (3)	−0.002 (3)
C6	0.075 (5)	0.084 (6)	0.055 (4)	−0.014 (5)	0.003 (4)	−0.012 (4)
C7	0.068 (5)	0.076 (6)	0.089 (6)	−0.003 (5)	−0.006 (4)	0.001 (5)
C8	0.068 (5)	0.102 (7)	0.061 (5)	0.000 (5)	−0.007 (4)	−0.014 (5)
C9	0.053 (3)	0.045 (3)	0.045 (3)	0.009 (3)	0.003 (3)	0.007 (3)
C10	0.054 (4)	0.069 (5)	0.073 (4)	0.005 (4)	0.017 (3)	0.014 (4)
C11	0.047 (3)	0.062 (4)	0.072 (4)	−0.015 (3)	0.010 (3)	0.003 (4)
O1	0.081 (4)	0.067 (4)	0.120 (5)	0.011 (3)	0.037 (3)	0.010 (4)
O2	0.068 (3)	0.053 (3)	0.074 (3)	−0.002 (3)	0.009 (2)	0.002 (3)
O3	0.059 (3)	0.043 (2)	0.074 (3)	−0.012 (2)	0.018 (2)	−0.011 (2)
O4	0.046 (2)	0.053 (3)	0.062 (3)	−0.011 (2)	0.009 (2)	−0.002 (2)
O5	0.082 (3)	0.066 (3)	0.052 (2)	−0.022 (3)	0.003 (2)	0.015 (3)
C12	0.062 (4)	0.061 (4)	0.046 (4)	−0.003 (4)	0.022 (3)	−0.005 (3)
C13	0.060 (4)	0.051 (4)	0.048 (3)	0.005 (4)	0.016 (3)	0.003 (3)
C14	0.041 (3)	0.048 (4)	0.042 (3)	0.007 (3)	0.014 (3)	0.006 (3)
C15	0.046 (3)	0.036 (3)	0.061 (4)	0.005 (3)	0.020 (3)	−0.004 (3)

### Geometric parameters (Å, °)

**Table:**

F1—C1	1.309 (14)	C7—C8	1.304 (13)
F3—C1	1.352 (13)	C8—H8A	0.9300
F2—C1	1.589 (14)	C9—C10	1.517 (8)
F1'—C1	1.436 (13)	C9—H9A	0.9800
F2'—C1	1.219 (13)	C10—H10A	0.9600
F3'—C1	1.167 (12)	C10—H10B	0.9600
F4—C2	1.395 (13)	C10—H10C	0.9600
F5—C2	1.274 (12)	C11—H11A	0.9600
F6—C2	1.404 (11)	C11—H11B	0.9600
F4'—C2	1.405 (14)	C11—H11C	0.9600
F5'—C2	1.474 (13)	O1—C12	1.237 (9)
F6'—C2	1.313 (15)	O2—C12	1.290 (8)
N—C11	1.480 (7)	O2—H2A	0.8200
N—C9	1.523 (7)	O3—C14	1.394 (7)
N—H0A	0.8600	O3—H3A	0.8200
C1—C7	1.495 (11)	O4—C15	1.213 (7)
C2—C3	1.392 (13)	O5—C15	1.236 (7)
C3—C4	1.336 (9)	O5—H5A	0.8200
C3—C8	1.447 (11)	C12—C13	1.463 (9)
C4—C5	1.432 (10)	C13—C14	1.530 (8)
C4—H4A	0.9300	C13—H13A	0.9700
C5—C6	1.315 (9)	C13—H13B	0.9700
C5—C9	1.492 (8)	C14—C15	1.543 (8)
C6—C7	1.441 (10)	C14—H14A	0.9800
C6—H6A	0.9300		
			
C11—N—C9	112.7 (5)	C5—C6—C7	121.6 (8)
C11—N—H0A	123.6	C5—C6—H6A	119.2
C9—N—H0A	123.6	C7—C6—H6A	119.2
F3'—C1—F2'	102.5 (11)	C8—C7—C6	119.4 (8)
F3'—C1—F1	128.3 (11)	C8—C7—C1	122.9 (8)
F2'—C1—F3	123.5 (10)	C6—C7—C1	117.6 (8)
F1—C1—F3	122.1 (10)	C7—C8—C3	120.8 (8)
F3'—C1—F1'	114.7 (11)	C7—C8—H8A	119.6
F2'—C1—F1'	94.2 (9)	C3—C8—H8A	119.6
F1—C1—F1'	56.8 (8)	C5—C9—C10	114.4 (5)
F3—C1—F1'	80.8 (8)	C5—C9—N	112.1 (5)
F3'—C1—C7	116.5 (10)	C10—C9—N	108.0 (5)
F2'—C1—C7	116.5 (9)	C5—C9—H9A	107.3
F1—C1—C7	113.2 (9)	C10—C9—H9A	107.3
F3—C1—C7	117.9 (7)	N—C9—H9A	107.3
F1'—C1—C7	110.3 (8)	C9—C10—H10A	109.5
F3'—C1—F2	59.7 (8)	C9—C10—H10B	109.5
F2'—C1—F2	56.9 (8)	H10A—C10—H10B	109.5
F1—C1—F2	96.9 (8)	C9—C10—H10C	109.5
F3—C1—F2	97.0 (8)	H10A—C10—H10C	109.5
F1'—C1—F2	144.0 (8)	H10B—C10—H10C	109.5
C7—C1—F2	102.4 (8)	N—C11—H11A	109.5
F5—C2—F6'	77.4 (9)	N—C11—H11B	109.5
F5—C2—C3	115.3 (9)	H11A—C11—H11B	109.5
F6'—C2—C3	130.1 (9)	N—C11—H11C	109.5
F5—C2—F4	106.1 (10)	H11A—C11—H11C	109.5
F6'—C2—F4	110.7 (10)	H11B—C11—H11C	109.5
C3—C2—F4	110.8 (10)	C12—O2—H2A	109.5
F5—C2—F6	103.2 (10)	C14—O3—H3A	109.5
C3—C2—F6	109.2 (9)	C15—O5—H5A	109.5
F4—C2—F6	112.1 (8)	O1—C12—O2	120.8 (7)
F5—C2—F4'	116.0 (10)	O1—C12—C13	121.8 (7)
F6'—C2—F4'	91.5 (9)	O2—C12—C13	117.4 (7)
C3—C2—F4'	119.0 (10)	C12—C13—C14	111.8 (5)
F6—C2—F4'	88.8 (8)	C12—C13—H13A	109.3
F6'—C2—F5'	104.7 (10)	C14—C13—H13A	109.3
C3—C2—F5'	107.4 (9)	C12—C13—H13B	109.3
F4—C2—F5'	81.7 (8)	C14—C13—H13B	109.3
F6—C2—F5'	132.1 (11)	H13A—C13—H13B	107.9
F4'—C2—F5'	99.5 (9)	O3—C14—C13	107.7 (4)
C4—C3—C2	124.2 (8)	O3—C14—C15	115.0 (5)
C4—C3—C8	118.5 (8)	C13—C14—C15	109.4 (5)
C2—C3—C8	116.8 (8)	O3—C14—H14A	108.2
C3—C4—C5	121.2 (7)	C13—C14—H14A	108.2
C3—C4—H4A	119.4	C15—C14—H14A	108.2
C5—C4—H4A	119.4	O4—C15—O5	126.3 (6)
C6—C5—C4	118.4 (7)	O4—C15—C14	117.3 (5)
C6—C5—C9	122.5 (7)	O5—C15—C14	116.3 (5)
C4—C5—C9	119.1 (6)		
			
F5—C2—C3—C4	−8.6 (14)	F2—C1—C7—C8	63.3 (12)
F6'—C2—C3—C4	86.2 (14)	F3'—C1—C7—C6	−56.5 (14)
F4—C2—C3—C4	−129.1 (9)	F2'—C1—C7—C6	−177.7 (11)
F6—C2—C3—C4	107.0 (10)	F1—C1—C7—C6	138.1 (10)
F4'—C2—C3—C4	−153.3 (8)	F3—C1—C7—C6	−13.7 (14)
F5'—C2—C3—C4	−41.5 (11)	F1'—C1—C7—C6	76.5 (10)
F5—C2—C3—C8	179.6 (8)	F2—C1—C7—C6	−118.7 (8)
F6'—C2—C3—C8	−85.6 (13)	C6—C7—C8—C3	−0.6 (14)
F4—C2—C3—C8	59.1 (11)	C1—C7—C8—C3	177.4 (8)
F6—C2—C3—C8	−64.8 (10)	C4—C3—C8—C7	2.0 (13)
F4'—C2—C3—C8	34.8 (13)	C2—C3—C8—C7	174.3 (9)
F5'—C2—C3—C8	146.6 (8)	C6—C5—C9—C10	−128.2 (7)
C2—C3—C4—C5	−174.3 (8)	C4—C5—C9—C10	49.9 (8)
C8—C3—C4—C5	−2.6 (11)	C6—C5—C9—N	108.5 (7)
C3—C4—C5—C6	1.8 (11)	C4—C5—C9—N	−73.5 (7)
C3—C4—C5—C9	−176.3 (6)	C11—N—C9—C5	−66.8 (7)
C4—C5—C6—C7	−0.3 (11)	C11—N—C9—C10	166.3 (5)
C9—C5—C6—C7	177.8 (7)	O1—C12—C13—C14	−75.2 (9)
C5—C6—C7—C8	−0.3 (13)	O2—C12—C13—C14	103.5 (7)
C5—C6—C7—C1	−178.4 (8)	C12—C13—C14—O3	−55.0 (7)
F3'—C1—C7—C8	125.5 (13)	C12—C13—C14—C15	70.6 (7)
F2'—C1—C7—C8	4.3 (17)	O3—C14—C15—O4	−167.3 (5)
F1—C1—C7—C8	−39.9 (15)	C13—C14—C15—O4	71.4 (6)
F3—C1—C7—C8	168.3 (10)	O3—C14—C15—O5	9.4 (7)
F1'—C1—C7—C8	−101.5 (12)	C13—C14—C15—O5	−111.9 (6)

### Hydrogen-bond geometry (Å, °)

**Table:**

*D*—H···*A*	*D*—H	H···*A*	*D*···*A*	*D*—H···*A*
N—H0A···O4^i^	0.86	2.37	2.914 (7)	122
N—H0A···O3^ii^	0.86	2.20	2.887 (7)	137

Symmetry codes: (i) *x*−1, *y*, *z*; (ii) −*x*+1, *y*+1/2, −*z*+1.
